# Supplementary material for: Effect of Team-Based Learning Interventions on the Learning Competency of Korean Nursing Students: A Systematic Review and Meta-Analysis
Source: Behav Sci (Basel). 2025 Mar 19;15(3):390. doi: 10.3390/bs15030390 (PMC11939142; doi:10.3390/bs15030390)
Supplement: Supplementary file 1 [file behavsci-15-00390-s001.zip › behavsci-3482736-supplementary/behavsci-3482736-supplementary File S1.pdf]

Supplementary Material S1: Systematic Review Protocol

Title:
Effect of Team-Based Learning Interventions on the Learning Competency of Nursing Students: A Systematic Review and Meta-Analysis

1. Background and Rationale
Team-Based Learning (TBL) is an active learning strategy that fosters student engagement, collaboration, and problem-solving skills. It has been widely implemented in medical and nursing education as an alternative to traditional lecture-based teaching. Given the increasing application of TBL in nursing programs, there is a need to synthesize existing evidence on its impact on nursing students' learning competencies.
This systematic review and meta-analysis aim to evaluate the effectiveness of TBL interventions in improving key learning competencies of undergraduate nursing students, including **communication ability, self-efficacy, learning ability, and problem-solving skills**. The findings from this study will contribute to evidence-based recommendations for integrating TBL into nursing education curricula.

2. Objectives
The objectives of this systematic review and meta-analysis are:
- To systematically evaluate the effect of **TBL interventions** on the **learning competencies** of undergraduate nursing students.
- To conduct a **meta-analysis** to quantify the impact of TBL compared to traditional teaching methods.
- To perform **subgroup analyses** to explore heterogeneity based on intervention characteristics (e.g., duration, type of TBL, assessment method).
-

3. Methods
3.1 Eligibility Criteria (PICO Framework)
- **Population:** Undergraduate nursing students participating in TBL-based courses.
- **Intervention:** Implementation of a **TBL** program in nursing education.
- **Comparison:** Traditional educational methods (e.g., lecture-based learning) or no intervention.
- **Outcomes:** Enhancement of learning competencies, specifically **communication ability, self-efficacy, learning ability, and problem-solving skills**.

3.2 Study Design
This review includes the following study designs:
- **Randomized Controlled Trials (RCTs)**
- **Quasi-experimental studies** with control groups
- **Pre-post intervention studies**

Studies without control groups, qualitative studies, and reviews were excluded.
3.3 Search Strategy
A comprehensive literature search was conducted using the following electronic databases:
- **Medline/PubMed**
- **Embase**
- **Cochrane Library**
- **CINAHL Complete**
- **Google Scholar**

The literature search was conducted over three months, including preliminary searches and strategy refinement, with the **final systematic search performed from October 1 to October 3, 2023**. Following **Cochrane Library guidelines**, a short final search period (1–3 days) was used to ensure **study reproducibility** while capturing the most recent publications.
Search Terms Used
Variations of "team-based learning" and "nursing education" were used, including:
- **"(team AND based AND learning) AND (nursing AND education)"**
- **"team-based learning" AND "nursing education"**
- **"TBL" AND "nursing education"**

To **increase search sensitivity** and reduce **publication bias**, we also:

- Reviewed **reference lists** of retrieved studies.
- Included **grey literature sources** (conference proceedings, unpublished dissertations, and preprints).
- Conducted **manual searches** to supplement database results.

Following **PRISMA (Preferred Reporting Items for Systematic Reviews and Meta-Analyses) guidelines**, the search was designed to be comprehensive, ensuring the incorporation of grey literature to enhance search sensitivity and reduce publication bias. However, this detail was not explicitly mentioned in the manuscript. To address this, we have **revised the manuscript** to provide a clearer explanation of our search strategy, including the incorporation of **grey literature sources**.

3.4 Study Selection and Data Extraction

Two independent reviewers conducted the study selection process. Titles and abstracts were screened to remove irrelevant studies, and full-text assessments were performed based on the eligibility criteria. Disagreements were resolved through discussion or consultation with a third reviewer.

Data Extraction Items:

- **Study characteristics:** Authors, publication year, country, study design.
- **Participant characteristics:** Sample size, demographic details.
- **Intervention details:** TBL format, duration, frequency, assessment methods.
- **Outcome measures:** Learning competencies measured (communication ability, self-efficacy, learning ability, problem-solving skills), statistical data (means, standard deviations, effect sizes).

Data were extracted using a standardized form and entered into Microsoft Excel for further analysis.

3.5 Risk of Bias and Quality Assessment

The risk of bias for included studies was assessed independently by two reviewers using the following tools:

- **Cochrane Risk of Bias (RoB) Tool** for RCTs.
- **Risk of Bias in Non-Randomized Studies (ROBINS-I)** for quasi-experimental studies.

The studies were categorized as having low, moderate, or high risk of bias based on selection bias, performance bias, detection bias, attrition bias, and reporting bias.

3.6 Data Synthesis and Meta-Analysis

- **Effect sizes** were calculated using **Standardized Mean Differences (SMDs)**.
- A **random-effects model** was applied due to anticipated heterogeneity in study designs and interventions.
- **Heterogeneity** was assessed using **I^2 statistics** and **Q-tests**.
- **Subgroup analyses** were conducted based on study design, intervention duration, and learning competency outcomes.
- A **sensitivity analysis** was performed to test the robustness of findings by excluding studies with a high risk of bias.
-

3.7 Publication Bias Assessment

Publication bias was evaluated using:

- **Funnel plots** to visually inspect asymmetry.
- **Egger's regression test** to statistically assess small-study effects.
-

4. Ethical Considerations

As this study is a systematic review of previously published data, ethical approval was not required. No identifiable patient data were used in the analysis.

5. Amendments and Updates

- This protocol follows **PRISMA 2020 guidelines**.
- Any amendments made during the review process will be documented accordingly and reported in the final manuscript.
