# Supplementary material for: Effect of Team-Based Learning Interventions on the Learning Competency of Korean Nursing Students: A Systematic Review and Meta-Analysis
Source: Behav Sci (Basel). 2025 Mar 19;15(3):390. doi: 10.3390/bs15030390 (PMC11939142; doi:10.3390/bs15030390)
Supplement: Supplementary file 1 [file behavsci-15-00390-s001.zip › behavsci-3482736-supplementary/behavsci-3482736-supplementary.pdf]

Supplementary Materials. Final List of 12 Studies Included in the Meta-Analysis

1. (Cho & Kweon, 2017) Cho, Y. H., & Kweon, Y. R. (2017). Effects of team-based learning on communication competence for undergraduate nursing students. *Journal of Korean Academy of Psychiatric and Mental Health Nursing*, *26*(1), 101-110. https://doi.org/10.12934/jkpmhn.2017.26.1.101
2. (Kim & Kim, 2016) Kim, S. O., & Kim, S. M. (2016). Effects of team-based learning applying for nursing students on critical thinking ability, problem solving ability and communication ability. *Journal of the Korean Data Analysis Society*, *18*(2), 1151-1161.
3. (Kim, 2016) Kim, H. R., Song, Y., Lindquist, R., & Kang, H. Y. (2016). Effects of team-based learning on problem-solving, knowledge and clinical performance of Korean nursing students. Nurse education today, 38, 115–118. https://doi.org/10.1016/j.nedt.2015.12.003
4. (Ko & Kim, 2017) Ko, E., & Kim, H. Y. (2017). Effects of simulation-based education combined team-based learning on self-directed learning, communication skills, nursing performance confidence and team efficacy in nursing students. *Journal of Korean Academy of Fundamentals of Nursing*, *24*(1), 39-50. http://dx.doi.org/10.7739/jkafn.2017.24.1.39
5. (Kim & Lee, 2018) Kim, J. Y., & Lee, M. K. (2018). Effect of team-based learning using reflection journal on pregnancy nursing course for nursing students. *Korean Journal of Women Health Nursing*, *24*(4), 404-413. https://doi.org/10.4069/kjwhn.2018.24.4.404
6. (Kim, 2019) Kim, Y. J. (2019). The Effects of Team-Based Learning (TBL) for Edutainment Activation in Nursing Students. *Journal of Korea Entertainment Industry Association*, *13*(7), 397-408. https://doi.org/10.21184/jkeia.2019.10.13.7.397
7. (Kim, 2020) Kim, H. J. (2020). The effects of pre-briefing team-based learning in standardized patients simulation. *Journal of Digital Convergence*, *18*(8), 271-279. https://doi.org/10.14400/JDC.2020.18.8.271
8. (Lee, 2018) Lee, S. H. (2018). Effect of practical delivery-nursing simulation education on team-based learning on the nursing knowledge, self-efficacy, and clinical competence of nursing students. *Korean Journal of Women Health Nursing*, *24*(2), 150-162. https://doi.org/10.4069/kjwhn.2018.24.2.150
9. (Lee, 2018) Lee, K. E. (2018). Effects of team-based learning on the core competencies of nursing students: A quasi-experimental study. *Journal of Nursing Research*, *26*(2), 88-96. http://dx.doi.org/10.1097/jnr.0000000000000259
10. (Oh, 2015) Oh, H. S. (2015). The effects of team-based learning on outcome based nursing education. *Journal of digital Convergence*, *13*(9), 409-418. http://dx.doi.org/10.14400/JDC.2015.13.9.409
11. (Yoon & Lee, 2018) Yoon, J. H., & Lee, E. J. (2018). The effect of team based simulation learning using SBAR on critical thinking and communication clarity of nursing students. *Journal of the Korea Academia-Industrial cooperation Society*, *19*(9), 42-49. https://doi.org/10.5762/KAIS.2018.19.9.42
12. (Yang, 2019) Yang, S. Y. (2019). Effects of team-based problem-based learning combined with smart education: a focus on high-risk newborn care. *Child Health Nursing Research*, *25*(4), 507-517. https://doi.org/10.4094/chnr.2019.25.4.507
